# Epidemiology, genetics and treatment of multiple myeloma and precursor diseases

**DOI:** 10.1002/ijc.33762

**Published:** 2021-08-30

**Authors:** Kari Hemminki, Asta Försti, Richard Houlston, Amit Sud

**Affiliations:** ^1^ Biomedical Center, Faculty of Medicine Charles University in Pilsen Pilsen Czech Republic; ^2^ Division of Cancer Epidemiology German Cancer Research Center (DKFZ) Heidelberg Germany; ^3^ Hopp Children's Cancer Center (KiTZ) Heidelberg Germany; ^4^ Division of Pediatric Neurooncology German Cancer Research Center (DKFZ), German Cancer Consortium (DKTK) Heidelberg Germany; ^5^ Division of Genetics and Epidemiology The Institute of Cancer Research London UK; ^6^ The Department of Haemato‐Oncology The Royal Marsden Hospital NHS Foundation Trust London UK

**Keywords:** clinical presentation, plasma cell disease, risks factors, survival, treatment

## Abstract

Multiple myeloma (MM) is a hematological malignancy caused by the clonal expansion of plasma cells. The incidence of MM worldwide is increasing with greater than 140 000 people being diagnosed with MM per year. Whereas 5‐year survival after a diagnosis of MM has improved from 28% in 1975 to 56% in 2012, the disease remains essentially incurable. In this review, we summarize our current understanding of MM including its epidemiology, genetics and biology. We will also provide an overview of MM management that has led to improvements in survival, including recent changes to diagnosis and therapies. Areas of unmet need include the management of patients with high‐risk MM, those with reduced performance status and those refractory to standard therapies. Ongoing research into the biology and early detection of MM as well as the development of novel therapies, such as immunotherapies, has the potential to influence MM practice in the future.

**Abbreviations**
ASCT: autologous stem cell transplantation
CLL: chronic lymphocytic leukemia
FLC: free light chain
GWAS: genome‐wide association study
Ig: immunoglobulin
IMWG: International Myeloma Working Group
ISS: International staging system
MDE: myeloma defining event
MGUS: monoclonal gammopathy of unknown significance
MM: multiple myeloma
OS: overall survival
PETHEMA: Programa Espanol de Tratamientosen Hematologia
SEER: Surveillance, Epidemiology and End Results
SM: smoldering myeloma
SNP: single nucleotide polymorphism
TTP: time to progression
VRD: bortezomib, lenalidomide, dexamethasone
VTD: bortezomib, thalidomide, dexamethasone

## 1 INTRODUCTION

Multiple myeloma (MM) is an incurable hematological malignancy caused by the clonal expansion of plasma cells. The malignant plasma cells generally reside in the bone marrow and produce an abnormal antibody (M‐protein).^1^ Over 140 000 cases of MM are diagnosed worldwide per year with a lifetime risk of MM in economically developed countries of 0.6% to 1%.^2^, ^3^, ^4^

The first case reports of MM appeared in the medical literature in the 1840s.^5^, ^6^, ^7^ In 1848, William Macintyre and Henry Bence Jones described an abnormal protein in the urine of a patient with MM and, in 1889, Otto Kahler described the archetypical clinical features.^8^, ^9^ Swedish scientist Arne Tiselius developed electrophoretic isolation of serum proteins in the 1930s and in 1961, another Swedish scientist, Jan Waldenström, described the pathognomonic monoclonal M‐protein.^10^ Since these landmark discoveries, our understanding of the biological basis and management of MM has progressed leading to improvements in survival.

## 2 CLINICAL PRESENTATION, INVESTIGATIONS AND STAGING

The most common presenting signs and symptoms of MM are anemia, bone pain, renal dysfunction, fatigue, hypercalcemia, infection and weight loss.^11^ Less common features include extradural spinal cord compression (due to extramedullary plasmacytoma or a bone fragment due to a vertebral body fracture), hepatomegaly, splenomegaly and hyperviscosity.^11^ The majority of MM patients have a M‐protein generated by the clonal plasma cell population. Of the different types of paraprotein produced, IgG accounts for 52% of cases, IgA 21%, light chain 16% and IgD, biclonal and IgM each account for <5% of cases. Serum protein electrophoresis will identify the abnormal protein in 80% of cases and serum protein immunofixation increases the sensitivity.^11^ A serum‐free light chain assay or urinary protein electrophoresis and immunofixation increase the sensitivity further, particularly as it identifies light chain‐only disease.^11^, ^12^, ^13^ Newer techniques based on mass spectrometry are emerging which have a number of clinical and analytical advantages when compared to serum protein electrophoresis.^14^ About 6.5% of MM cases are thought to be oligosecretory or nonsecretory.^11^

Monoclonal gammopathy of unknown significance (MGUS) is the progenitor disease of MM, where the patient is often asymptomatic and the M‐protein is typically present at a lower concentration than in MM (Table 1).^15^, ^16^ The annual risk of progression of MGUS to MM is 1%.^17^ The prevalence of MGUS increases with age and is detectable in 1.7% of those aged 50 to 59 years and 6% of individuals aged over 80 years.^18^ Smoldering myeloma (SM) represents an intermediate clinical stage between MGUS and MM (Table 1).^19^ Other diseases related to MM, termed plasma cell dyscrasias, include light chain AL amyloidosis, and plasma cell leukemia.^19^ When IgM MGUS progresses to symptomatic disease, it typically results in Waldenström's macroglobulinemia (a mature B‐cell neoplasm), although rare cases of IgM MM have been reported.

**TABLE 1:** Summary of diagnostic criteria for MGUS, SM and MM

	Monoclonal protein and clonal bone marrow plasma cells	Myeloma defining event (biomarker of malignancy^a^ or end‐organ damage)
MGUS	Serum monoclonal protein <30 g/L and urinary monoclonal protein <500 mg per 24 hours and clonal bone marrow plasma cells <10%	No
SM	Serum monoclonal protein ≥30 g/L or urinary monoclonal protein ≥500 mg per 24 hours or clonal bone marrow plasma cells 10% to 60%	No
MM	Clonal bone marrow plasma cells ≥10% or biopsy‐proven plasmacytoma	Yes

Abbreviations: MGUS, monoclonal gammopathy of undetermined significance; SM smoldering myeloma; MM, multiple myeloma. FLC ratio, involved versus uninvolved serum‐free light chain ratio.

^a^ Biomarker of malignancy: ≥60% clonal bone marrow plasma cells; ≥100 FLC ratio (absolute level of the involved light chain is at least 100 mg/L); >1 lesion by magnetic resonance imaging (≥5 mm in size). End‐organ damage (due to myeloma): hypercalcemia (serum calcium >0.25 mmol/L [>1 mg/dL] higher than the upper limit of normal or >2.75 mmol/L [>11 mg/dL]); renal insufficiency (creatinine clearance <40 mL per minute or serum creatinine >177 mol/L [>2 mg/dL]); anemia (hemoglobin >20 g/L below the lowest limit of normal or hemoglobin <100 g/L); bone lesions: one or more osteolytic lesion on skeletal radiography, CT or PET/CT. If bone marrow has <10% clonal plasma cells, more than one bone lesion is required to distinguish from solitary plasmacytoma with minimal marrow involvement.

All patients with suspected MM require cross‐sectional imaging to assess for myeloma‐related bone disease and extramedullary disease.^20^, ^21^ Due to the higher sensitivity when compared to conventional skeletal surveys, whole‐body low‐dose CT is the current standard first‐line imaging modality when investigating SM, MM, relapse or prior to maintenance therapy in the absence of previous FDG‐avid disease.^20^, ^21^, ^22^ PET‐CT is recommended when investigating extramedullary solitary plasmacytomas, as an alternative in suspected MM or for revaluation of previous FDG‐avid disease prior to maintenance therapy. Whole‐body diffusion‐weighted MRI is currently recommended where low‐dose CT does not demonstrate disease where MM is suspected, is inconclusive or in the reassessment of disease after treatment, although this is becoming the primary imaging modality in some centers.^20^, ^21^

Quantification of plasma cell infiltration is performed using morphological assessment of a bone marrow aspirate or biopsy with or without immunohistochemistry with antibodies to plasma cell associated antigens, such as CD138.^23^, ^24^ Whereas the quantity of plasma cells may be underestimated by flow cytometry compared to morphological assessment, the higher sensitivity of multicolor flow cytometry allows for the detection of small numbers of plasma cells, which may be missed by morphological or immunohistochemical evaluation.^25^, ^26^ Cytogenetic analysis by fluorescence in situ hybridization on purified clonal plasma cells should include tests for the high‐risk cytogenetic abnormalities including t(4;14), t(14;16) and del(17p).^27^

Traditionally, a diagnosis of MM required the presence of end‐organ damage, which for diagnostic purposes, took the form of the CRAB criteria (C = elevated calcium, R = renal failure, A = anemia, B = bone lesions). In 2014, the International Myeloma Working Group (IMWG) revised the diagnostic criteria for MM and plasma cell dyscrasias which is summarized in Table 1.^19^ One major change was the addition of myeloma defining events (MDEs) to the traditional features of end‐organ damage when making a diagnosis of active MM. The aim of this change was to identify and treat individuals with a diagnosis of SM and a >80% probability of progression to end‐organ damage within 2 years.^19^, ^28^ Recently, whole‐genome sequencing (WGS) has been utilized to identify individuals with MM precursor diseases with low disease burden at a high‐risk of progression.^29^ Confirmation of such findings in larger studies is required along with the assessment of the risk discrimination afforded by WGS.

The factors which influence clinical outcomes of patients with MM can be divided into characteristics related to the tumor and those related to the patient. The MM International Staging System (ISS) formalizes such features and is based on serum albumin and β_2_‐microglobulin (β_2_M) concentrations.^30^ This staging system has been refined as the Revised International Staging System (R‐ISS), which incorporates information concerning somatic genetics, namely t(4;14), t(14;16) and del(17p), and lactate dehydrogenase concentration (Table 2).^31^ There is no unified definition of high‐risk myeloma (patients who experience early disease progression and death) but characteristics used include gene expression profiling, ISS Stage III disease, extramedullary disease or plasma cell leukemia or the presence of del(17p), 1q21 gain, t(4;14), t(14;16).^32^, ^33^ Additional somatic genomic classifiers such as biallelic *TP53* inactivation or amplification (≥4 copies) of CKS1B (1q21) have been found to add further discrimination beyond the R‐ISS.^33^, ^34^

**TABLE 2:** The International Myeloma Working Group revised international staging system (R‐ISS)^31^

Stage	R‐ISS	5‐year OS
I	Serum albumin >3.5 g/dL Serum β_2_‐microglobulin <3.5 mg/L No high‐risk cytogenetic features Normal serum lactate dehydrogenase level	82%
II	Neither Stage I or III	62%
III	Serum β_2_‐microglobulin >3.5 mg/L and high‐risk cytogenetics (t(4;14), t(14;16), del(17p)) or elevated serum lactate dehydrogenase level	40%

Abbreviation: OS, overall survival.

## 3 EPIDEMIOLOGY OF MYELOMA

The incidence of MM varies by sociodemographic status with the highest rate in high‐income countries (4‐6 per 100 000) and a 10‐fold difference between countries with the lowest and the highest rates.^4^ From 1990 to 2016, the incidence of MM has increased by 126%.^4^ This increase is largely due to a rise in age‐specific incidence rate, an aging population and population growth. Under‐reporting of cases at the start of cancer registries, changes to the diagnostic classification as well as resource‐stratified guidelines are likely to have contributed to the increase in the number of cases.^35^ In countries with a high sociodemographic index, mortality from MM peaked in the year 2000 whereas, in most other countries, mortality from MM continues to increase.^4^ Such trends can be explained by disproportionate improvements in care in regions with a high sociodemographic status.^4^, ^36^

**FIGURE 1:**
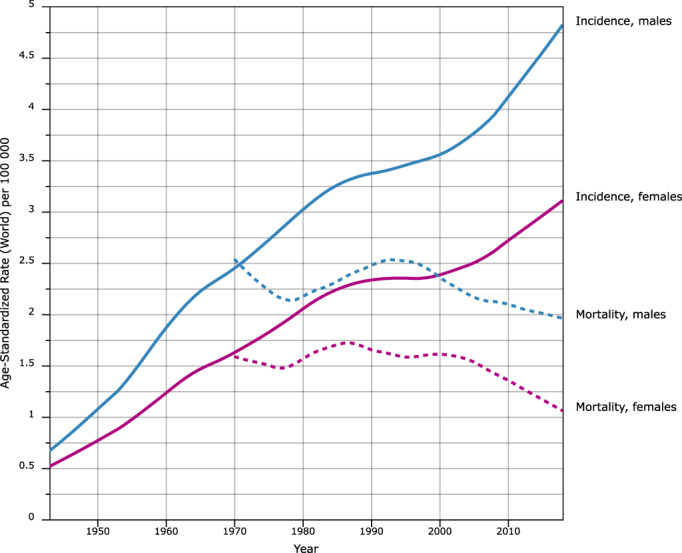
Incidence (from 1943, solid lines) and mortality (from 1950, broken lines) in multiple myeloma in Denmark to 2016. Lines corresponding to men are blue whereas lines corresponding to women are magenta. The rates are adjusted to the world standard population. The data are from the NORDCAN database from the International Agency for Research on Cancer [Color figure can be viewed at wileyonlinelibrary.com]

Figure 1 shows the incidence and mortality rates (adjusted to world standard age structure) for Denmark. The Danish data are unique in that they originate from the first national cancer registry in the world (established in 1943). The other aspect is that Copenhagen, the capital of Denmark, is located only 30 km from the city of Malmö, where Jan Waldenström was working. Thus, it is likely that MM was a well‐known disease in Denmark. The incidence rates for men and women have increased 6‐fold in the 73‐year period through 2016 (Figure 1). The male rates are 50% higher than the female rates but the increase in both is parallel. Mortality rates are close to parallel, with two maxima, one in the early 1960s and the second around 1990. Until the early 1960s, the mortality rate from MM was higher than the incidence rate. Given death registration is independent of cancer registration, Figure 1 suggests that there was a large under‐registration of incident MM cases, which the death registrar was subsequently able to attribute to MM.

Under‐reporting of MM cases in Denmark becomes clearer when we review MM epidemiology in Jan Waldenström's side, Sweden (Figure S1). Whereas the sex proportions were identical to Denmark, the incidence peaked much earlier, for men before 1990 and for women in 1975; yet, the maximal incidence rates were not much different from those in Denmark 30 years later. As the death rates in the two countries were relatively parallel, it is likely that the under‐reporting in Denmark continued well into the 2000s. Even if we have no proof that our interpretation of under‐reporting is correct, the example reminds about the difficulties in interpreting observational epidemiological data between two neighboring countries, let alone on the global scale.

Changes in risk factors may provide an alternative explanation for variation in the incidence rates of MM. To date, known risk factors include ethnicity, family history and the presence of a precursor disease state (MGUS and SM). Environmental risk factors for MM and precursor diseases have been reviewed.^37^, ^38^ A 2.4‐fold increase in MGUS was found in US Vietnam War Veterans exposed to Agent Orange, although other confounding factors cannot be excluded as a cause for the increased risk observed.^39^ The study was followed up by analysis of serum levels of microRNAs (miRNAs) in the exposed soldiers providing evidence on TCDD disrupted miRNA homeostasis.^40^ Even pesticide use in agriculture has been associated with an increased risk of MGUS.^41^, ^42^ In an occupational health study from the Nordic countries, male and female farmers were the only population with an increased risk of MM (however relative risk of only 1.1).^43^ Firefighters engaged in the containment of the World Trade Center attacks had a 2‐fold increased risk of MGUS although other confounders cannot be excluded.^44^ An increased risk of MM has been reported among firefighters from the Nordic countries.^45^ Risk of senile cataract and glaucoma was increased in persons earlier diagnosed with MM, MGUS, AL amyloidosis and Waldenström's macroglobulinemia.^46^ The reason was suggested to be M‐protein‐related increase in blood viscosity disturbing protein structure of the lens of the eye which is exquisitely sensitive to protein aggregation; ambient protein concentration of the lens is the highest of any tissue and lens proteins are extremely long‐lived.^47^ Additionally, positive associations have been described for MM and immune related factors.^48^ Nevertheless, common risk factors of cancer, including cigarette smoking, obesity, socioeconomic level, educational background or radiation exposure (atomic bomb survivors) do not appear to play a role.^49^, ^50^, ^51^, ^52^, ^53^ Individuals of black ethnicity have a 2‐fold increased risk of MM when compared to white individuals, whereas the incidence of MM is markedly lower in Asians.^54^ Whereas there could be several explanations, it is not excluded that ethnic variations are the result of genetic differences between populations.

## 4 FAMILIAL RISKS

An inherited component to MM susceptibility was first suggested in the 1920s. Since then a number of families with multiple cases of MM and other plasma cell dyscrasias have been reported. The first systematic population studies emerged from Sweden in the early 2000s. According to these Swedish epidemiological studies, the familial risk of MM has been reported to be ~2.5.^55^, ^56^, ^57^ MM is associated with an elevated familial risk of its precursor disease MGUS.^58^, ^59^, ^60^ An increased risk has also been reported for MM with other B‐cell malignancies such as chronic lymphoid leukemia, acute lymphoblastic leukemia and lymphoplasmacytic lymphoma/Waldenstöm's macroglobulinemia as well as the myeloproliferative neoplasms.^2^, ^55^, ^56^, ^61^ Table S1 shows significant associations, as relative risks, using data from a Swedish family study on MM.^62^ Significant associations with MM included colorectal, breast and prostate cancers and CLL, in addition to MM. The small excess familial risks with breast and prostate cancers may be an indication of shared risk factors between these cancers.^63^

## 5 GERMLINE GENETICS

Motivated by epidemiological studies demonstrating familial aggregation, there has been significant interest in identifying DNA sequence variants that predispose for MM. To date, no high penetrance susceptibility loci have been identified for MM.^64^, ^65^, ^66^ However, recent sequencing efforts have proposed novel candidates, most notably loss‐of‐function variants in *DIS3* and *KDM1A*.^67^, ^68^, ^69^, ^70^, ^71^

Support for polygenic susceptibility to MM has been provided by genome‐wide association studies (GWAS).^70^, ^72^, ^73^, ^74^, ^75^, ^76^ Figure S2 summarizes the most recent GWAS of MM risk comprising 9974 cases and 247 556 controls of European ancestry. The 24 loci that reach genome‐wide significance account for 16% of the SNP heritability for MM in European populations. A number of these risk loci have been subject to functional analysis, such as 7p15.3 (*CDCA7L*) and 5q15 (*ELL2*).^76^, ^77^, ^78^, ^79^, ^80^, ^81^ The lead SNP at 7p15.3 creates a new binding site for the transcription factor IRF4 and alters *CDCA7L* expression.^77^, ^78^ At 5q15, the risk SNPs are associated with reduced *ELL2* expression.^79^, ^80^ *ELL2* encodes a key component of the super elongation complex, which mediates rapid gene induction by suppressing transient pausing of RNA polymerase II. B cell‐lineage *ELL2* conditional knockout mice exhibit diminished humoral responses to immunization,^81^ and the same ELL2 allele that predisposes for MM also reduces IgA and IgG levels.^76^, ^82^ The arrow in Figure S2 marks the *CCND1* 870G>A polymorphism (11q13.3) that influences splicing of the *CCND1* mRNA and is associated with translocation t(11;14) MM. This provided the first evidence for genetic variation being a determinant of a specific somatic chromosomal aberration.^83^ Genome‐wide interaction and pathway‐based analysis revealed interactions with immune modulation and B‐cell development pathways.^84^ A systematic analysis of MM risk loci has provided evidence of the role of disrupted cell cycle signaling, apoptosis and autophagy in MM susceptiblity^70^, ^72^ (Figure S3).

A genetic correlation exists between MGUS and MM suggesting that the MM risk loci exert their biological effect, at least in part, before the establishment of MGUS and contribute to familial clustering.^58^, ^59^, ^60^, ^85^, ^86^ Moreover, the shared genetic risk factors observed between MM with other B‐cell malignancies such as AL amyloidosis and CLL suggests shared etiology and biology in oncogenesis.^87^, ^88^

## 6 SOMATIC GENETICS AND DISEASE BIOLOGY

**FIGURE 2:**
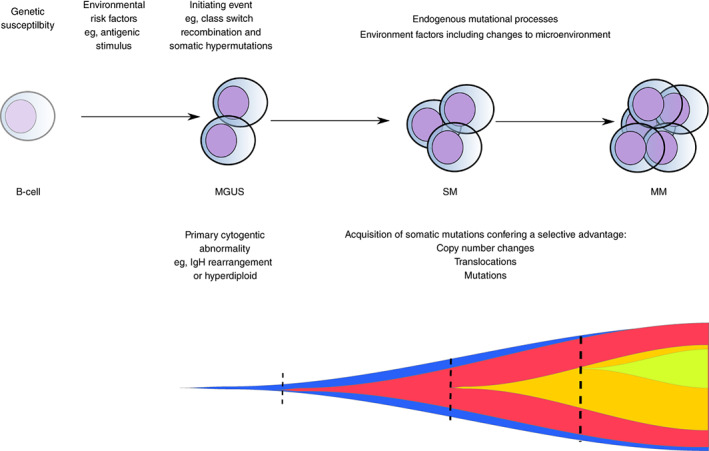
Model for the pathogenesis and evolutionary trajectory of multiple myeloma. MGUS, monoclonal gammopathy of undetermined significance; SM smoldering myeloma; MM, multiple myeloma. The fish plot demonstrates a model for the evolutionary trajectory of myeloma. The vertical dashed lines represent punctuated evolution which is characterized by the emergence of subclones that may become dominant. Static evolution occurs in‐between and represents the expansion of existing subclones under positive selection [Color figure can be viewed at wileyonlinelibrary.com]

The B‐cell malignancies arise from the unrestrained clonal expansion of B‐cells at different stages of maturation.^1^, ^89^ Conceptually, the development of MM can be thought of as an initiating transforming event occurring on the background of genetic susceptibility, the acquisition of additional somatic genetic events in the context of a microenvironment conducive to clonal expansion (Figure 2).

In the germinal center, the B‐cell receptor of a naïve B‐cell undergoes class‐switch‐recombination to alter the effector function of antibodies and somatic hypermutation to increase the affinity of the B‐cell receptor to a given antigen.^90^, ^91^, ^92^ Activation‐induced‐deaminase (AID) enzyme introduces DNA double‐strand breaks promoting both class‐switch‐recombination and somatic hypermutation.^93^ Given the majority of MM cases express class‐switched immunoglobulin heavy chain (IgH) constant regions with almost all demonstrating somatic hypermutation, the clonal plasma cells which characterize MM appear to expand from a postgerminal center B‐cell.^94^

The initial transforming event is thought to be an abnormal germinal center B‐cell response to an unknown antigenic stimulus. Consistent with large population‐based studies, reconstruction of the chronological activity of mutational signatures using sequencing data suggests the initial transforming event occurs in the second or third decade of life.^95^, ^96^

Approximately half of MGUS cases are likely caused by a primary translocation event that occurs at the time of immunoglobulin switch recombination.^97^ These translocations result in the juxtaposition of the IgH locus at 14q32 with an oncogene, the most common being 11q13 (*CCND1*), 6p21 (*CCND3*), 4p16.3 (FGFT3/*MMSET*), 16q23 (*MAF*) and 20q11 (*MAFB*).^98^ The resulting fusion causes dysregulation of the oncogene by placing it under the control of regulatory elements at the IgH locus.^99^, ^100^, ^101^, ^102^ The majority of the remaining MGUS cases are hyperdiploid, resulting in aneuploidy of chromosomes 3, 5, 7, 9, 11, 15 and 19.^103^ These gains in chromosome number appear to occur early but at multiple distinct time points.^104^

After the establishment of a clone with a primary cytogenetic abnormality, the acquisition of additional structural, copy number and single nucleotide variants results in a positive selection of a dominant clone as well as clonal heterogeneity.^104^, ^105^, ^106^, ^107^, ^108^, ^109^, ^110^, ^111^, ^112^, ^113^, ^114^, ^115^, ^116^, ^117^ Two models have been suggested to describe the evolutionary trajectory of progression. In the “static progression model”, the subclonal architecture is maintained as the disease advances suggesting progression to a clinical diagnosis reflects the time needed to accumulate a significant disease burden. In the “spontaneous evolution model”, a change in the subclonal composition is observed through the acquisition of additional mutations conferring a proliferative advantage to one of the subclones.^118^ Notable copy number changes and translocations include del(1p), del(11q), del(13q), del(17p), 1q gain and translocations involving *MYC*.^119^, ^120^, ^121^, ^122^ Point mutations occur most often in *NRAS*, *KRAS*, *BRAF*, *TENT5* and *CDKN2C*.^107^, ^123^ Pathways annotated by somatic genetic abnormalities include the RAS/MAPK signaling, DNA damage response and the NF‐κB pathway (Figure S3).^108^ The discovery of such somatic mutations has informed the development of targeted therapies such as MEK inhibitors and BRAF inhibitors.^124^, ^125^ Such targeted approaches are relatively new to MM, but based on experience in other cancers, will likely be challenged by multiple resistance mechanisms as well as clonal heterogeneity.^112^, ^126^

As well as genetic changes, alteration of gene expression through epigenetic dysregulation, for example, the abnormal histone methylation pattern observed with MMSET overexpression in t(4;14),^127^ contributes to the pathogenesis MM. Both genetic and epigenetic dysregulation converge on a number of biological processes including cell cycle perturbation and dysregulation of apoptosis.^128^, ^129^, ^130^, ^131^, ^132^, ^133^

In addition to the plasma cell clone, the microenvironment is reshaped in MM through induction of angiogenesis,^134^ suppression of antitumor immunity,^135^, ^136^ and modulation of plasma cell growth by bone marrow stromal cells.^137^

## 7 MANAGEMENT OF MGUS AND SMOLDERING MYELOMA

The annual risk of progression of MGUS to active disease is 1%.^17^ For SM, the annual risk of progression to MM is 10% for the first 5 years after a diagnosis, 3% for the subsequent 5 years, and 1% thereafter.^138^ However, risks vary within these groups and individuals with MGUS and SM should therefore undergo risk stratification to determine the risk of developing MM or an associated lymphoproliferative disease and to assist in determining the interval and location of follow‐up.^11^, ^139^, ^140^, ^141^ Currently used risk calculators include the Mayo clinic or PETHEMA calculator for MGUS and the PETHEMA or the revised Mayo clinic calculator (2/20/20; M‐protein >2 g/dL, bone marrow plasma cells >20%, involved/uninvolved free light chain [FLC] ratio >20) for SM.^142^, ^143^, ^144^ Using widely available tests that are reflective of clonal plasma cell burden, the revised Mayo clinic calculator for SM identifies three risk groups (no risk factors; median time to progression [TTP] = 110 months); intermediate risk (one risk factor; TTP = 68 months); and high risk (≥2 risk factor; TTP = 29 months). The addition of cytogenetic abnormalities (*MYC* abnormalities, t(4;14), t(14;16), +1q and/or del13q), MAPK pathway mutations and DNA repair pathway mutations may refine risk stratification of patients with SM.^113^, ^140^ Treating individuals with SM at high‐risk of progression, with the aim of preventing end‐organ damage, is currently of interest. Two approaches have been advocated, a low‐intensity clonal control approach,^145^, ^146^ or a high‐intensity clonal eradication approach.^147^ Whereas treating asymptomatic individuals with plasma cell disorders is theoretically attractive, concerns exist that early treatment adds cost and therapeutic burden in the absence of robust evidence supporting survival or a health‐related quality of life benefit.^148^

Care should be taken to identify individuals with MGUS with unexplained symptoms and signs as further investigation may identify patients with monoclonal gammopathies of clinical significance.^149^ Due to data demonstrating an increased risk of infection, individuals with MGUS, SM and MM in remission should be vaccinated against the influenza virus, pneumococci and hemophilus influenzae.^150^, ^151^ Vaccination against hepatitis A, hepatitis B, meningococcus, tetanus, diphtheria toxoids, acellular pertussis and herpes zoster is dependent on immune function, previous vaccinations and potential exposure.^151^ Vaccinations should ideally occur in the absence of active disease.^152^

There is significant interest in the early detection of plasma cell dyscrasias in the population. Initiatives such as the Iceland Screens Treats or Prevents Multiple Myeloma (iStopMM) study and the Promise study plan to identify individuals in the population with precursor clonal plasma cell disorders with the aim of understanding the natural history and biology of these diseases and ultimately treating individuals with high‐risk, asymptomatic disease.

## 8 MANAGEMENT OF MYELOMA

### 8.1 Historical perspective

Before the 1960s, the treatment of MM was directed toward the alleviation of symptoms rather than controlling the disease.^153^ Due to the cytotoxic effects, urethane was used in the middle of the 1900s until it was shown ineffective in 1966.^154^ By the late 1950s, another cytotoxic agent melphalan became available and when combined with prednisone (a steroid which in isolation reduced M‐protein levels) was the first combination therapy to produce objective response in MM.^155^, ^156^ With the aim of inducing complete remission through the use of larger doses of cytotoxic, high‐dose melphalan followed by autologous stem cell transplantation (ASCT) was first reported in a patient with MM at the Royal Marsden Hospital in 1983.^157^ Bart Barlogie successfully used thalidomide (used initially for the anti‐angiogenic properties) in 1997 to treat a patient with MM and the first clinical trial of its use was published in 1999.^158^, ^159^, ^160^ It was not until 2010 that CRBN was identified as a target of thalidomide and 2014 when IKZF1 and IKZF3 as the degradation targets of the CRL4^CRBN^ E3 ubiquitin ligase.^161^ Proteasome inhibitors were developed in the late 20th century with the aim of interfering with the ordered, temporal degradation of proteins responsible for cancer cell proliferation. In 2004, the first trial demonstrating the efficacy of the proteasome inhibitor Bortezomib in MM was published.^162^, ^163^, ^164^

### 8.2 General principles

**FIGURE 3:**
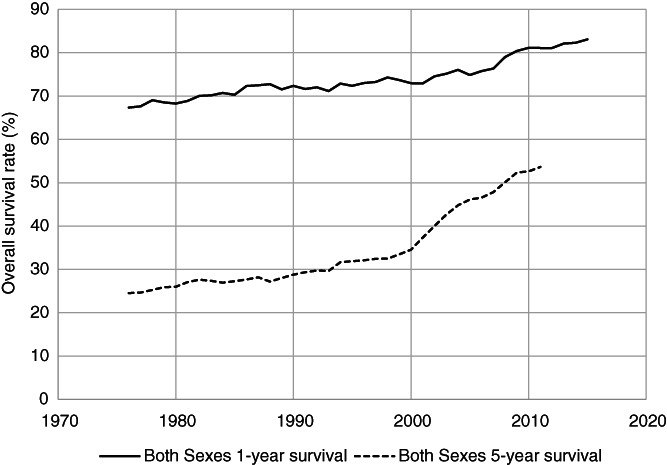
Trends in relative survival in multiple myeloma based on the US Surveillance, Epidemiology and End Results (SEER) database from 1975 through 2016, for both sexes and all ethnic groups. The upper graph is 1‐year and the lower 5‐year relative survival

Such developments in antimyeloma therapies have contributed to improvements in survival.^165^, ^166^, ^167^ Relative survival (ie, survival in MM patients compared to survival in the age‐adjusted background population) from the US Surveillance, Epidemiology and End Results (SEER) database is illustrated in Figure 3.^168^ In 1975, 1‐year survival in the SEER population (all ethnics) was 68% which improved to 84% in 2016. Five‐year survival has also increased from 28% in 1975 to 56% in 2012 with survival for men and women being similar. Such improvements in survival are also seen in European countries.^167^, ^169^, ^170^

At least seven different classes of agents have now been approved (Table 3). These agents are combined in doublet, triplet or quadruplet regimens, used with or without ASCT, or as continuous treatment. With such choice, defining optimal therapy at diagnosis and at each disease relapse, along with sequencing of such therapies, is challenging. Three drug regimens are most frequently used although two drug regimens have a role in certain clinical scenarios such as in frail patients.^171^, ^172^, ^173^, ^174^, ^175^ Aside from allogenic stem cell transplantation, which is not routinely performed,^176^ no treatment for MM is currently regarded as curative.

**TABLE 3:** Classes of drugs approved for use in multiple myeloma

Drug	Major mechanism of action	Administration route	Unwanted effects and cautions
Steroids			
Prednisolone	Glucocorticoid receptor agonist	Oral	Hypertension, infection, steroid‐induced diabetes, cataracts, adrenal suppression, avascular necrosis, myopathy, mood disturbance, sleep disturbance, gastrointestinal ulcer disease
Dexamethasone	Glucocorticoid receptor agonist	Oral, Intravenous	Hypertension, infection, steroid‐induced diabetes, cataracts, adrenal suppression, avascular necrosis, myopathy, mood disturbance, sleep disturbance, gastrointestinal ulcer disease
Alkylating agents			
Melphalan	Crosslinking of DNA and generation of double‐strand breaks	Oral, Intravenous	Myelosuppression, infection, mucositis, secondary malignancy, accumulation in renal failure
Cyclophosphamide	Crosslinking of DNA and generation of double‐strand breaks	Oral, Intravenous	Myelosuppression, infection, mucositis, secondary malignancy, hemorrhagic cystitis at high doses
Immunomodulatory drugs			
Thalidomide	Binds to CRBN inducing proteasomal degradation of IKZF1 and IKFZ3	Oral	Peripheral neuropathy, venous thromboembolism, somnolence, rash, teratogenic
Lenalidomide	Binds to CRBN inducing proteasomal degradation of IKZF1 and IKFZ3 (differing CRBN binding properties and degradation targets when compared to other immunomodulatory agents)	Oral	Myelosuppression, venous thromboembolism, diarrhea, constipation, rash, teratogenic, accumulation in renal failure, second cancers
Pomalidomide	Binds to CRBN inducing proteasomal degradation of IKZF1 and IKFZ3 (differing CRBN binding properties and degradation targets when compared to other immunomodulatory agents)	Oral	Bone marrow suppression, venous thromboembolism, rash, teratogenic
Proteasome inhibitors			
Bortezomib	First‐generation reversible boronic acid proteasome inhibitor	Subcutaneous, Intravenous	Peripheral neuropathy, thrombocytopenia, gastrointestinal toxicity, herpes zoster infection
Carfilzomib	Second‐generation irreversible tetrapeptide epoxyketone‐based proteasome inhibitor	Intravenous	Hypertension, cardiac failure, acute renal failure; thrombotic microangiopathy; cytopenia
Ixazomib	Reversible boronic acid proteasome inhibitor	Oral	Thrombocytopenia, gastrointestinal toxicity, rash, lower incidence of neuropathy compared to bortezomib
Histone deacetylase inhibitors			
Panobinostat	Pan‐deacetylase inhibitor	Oral	Thrombocytopenia, gastrointestinal toxicity
Nuclear export inhibitors			
Selinexor	Nuclear export inhibitor	Oral	Nausea, anorexia, diarrhea, hyponatremia, thrombocytopenia, fatigue
Anthracyclines			
Doxorubicin	Topoisomerase II inhibitor	Intravenous	Cardiac failure, myelosuppression, infection, second malignancy
Monoclonal antibodies			
Daratumumab	Humanized CD38‐targeting antibody	Subcutaneous, Intravenous	Infusion‐related reactions, interference with protein electrophoresis and blood group serological testing
Isatuximab	Chimeric CD38‐targeting antibody	Intravenous	Infusion‐related reactions, interference with protein electrophoresis and blood group serological testing
Elotuzomab	Humanized SLAMF7‐targeting antibody	Intravenous	Infusion related reactions, interference with protein electrophoresis
Belantamab Mafodotin	Afucosylated, humanized BCMA targeting antibody conjugated to a microtubule‐disrupting drug (monomethyl auristatin F)	Intravenous	Nausea, keratopathy, thrombocytopenia
CAR‐T cell therapy			
Idecabtagene Vicleucel (ide‐cel)	Transduction and infusion of autologous T cells with a lentiviral vector encoding a second‐generation CAR encoding an anti‐BCMA single‐chain variable fragment, a CD137 costimulatory motif and a CD3‐zeta signaling domain.	Intravenous	Cytokine release syndrome, immune effector cell‐associated toxicity, cytopenia

Abbreviations: CAR, chimeric antigen receptors; DNA, deoxyribonucleic acid.

Motivated by higher rates of complete response seen with new treatment approaches and the use of more sensitive techniques to measure disease (minimal residual disease) such as high throughput sequencing, the IMWG have recently revised the response categories used to assess the effect of treatment.^177^ Depth of response to treatment is correlated with improved patient outcomes, although this relationship is dependent on disease biology, therapy and time point of assessment.^178^, ^179^

**FIGURE 4:**
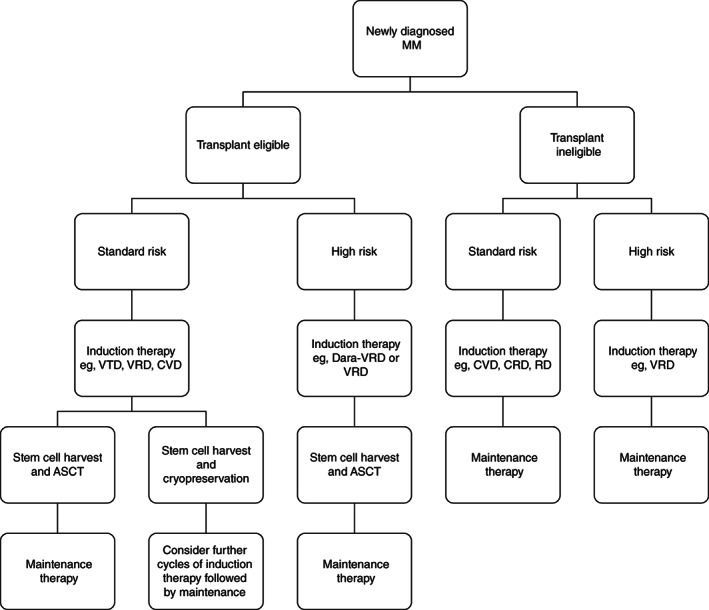
Treatment schema for newly diagnosed multiple myeloma. MM, multiple myeloma; VTD, bortezomib, thalidomide, dexamethasone; VRD, bortezomib, lenalidomide, dexamethasone; CVD, cyclophosphamide, bortezomib, dexamethasone; Dara‐VRd, daratumumab, bortezomib, lenalidomide, dexamethasone; CRD, cyclophosphamide, lenalidomide, dexamethasone; RD, lenalidomide, dexamethasone; ASCT, autologous stem cell transplantation. A guide to the current approach and possible regimens used to treat newly diagnosed multiple myeloma

After diagnosis and risk stratification, all patients should be assessed to determine eligibility for ASCT. When compared to chemotherapy alone, ASCT prolongs both progression‐free survival and overall survival and is performed immediately after induction therapy.^180^, ^181^, ^182^, ^183^ An general schema of the frontline management of MM is provided in Figure 4.

### 8.3 Patients eligible for autologous stem cell transplantation

Induction therapy is required before ASCT to reduce disease burden, improve symptoms and mitigate organ damage. The most common treatment regimens used contain bortezomib (eg, bortezomib, thalidomide, dexamethasone [VTD] or bortezomib, lenalidomide, dexamethasone [VRD]). Overall response rates with such therapy are generally >80%.^173^, ^174^, ^184^, ^185^ After induction therapy, usually 3 to 6 cycles, a stem cell harvest is performed. These stem cells are reinfused 1 to 2 days after high‐dose chemotherapy (usually melphalan). Many centers harvest sufficient stem cells to support a second ASCT, either as a tandem procedure (for high‐risk myeloma) or at relapse. The mortality associated with ASCT is 1% to 2% and is higher in individuals with co‐morbidities such as dialysis‐dependent renal failure.^186^ As the depth of response to induction therapy has improved with the use of novel therapies, the timing of ASCT has been debated.^184^, ^187^, ^188^

The benefit of consolidation (using the same therapy at induction post‐ASCT) is not currently clear.^188^, ^189^ Maintenance therapy, however, in the form of single‐agent lenalidomide, has shown a survival benefit and is now routinely practiced.^190^

### 8.4 Patients ineligible for autologous stem cell transplantation

Improvements in outcomes have been less pronounced for patients who are not eligible for ASCT. This is in part, related to poor performance status, co‐morbidities and low tolerance of multidrug regimens.^191^, ^192^ Improved supportive care, novel therapies and improved frailty assessment are now translating into improved outcomes in this patient population.^193^ Current regimens used range from bortezomib‐based triplet therapy possibly with dose attenuation,^194^, ^195^ lenalidomide‐based triplet therapy,^196^ and doublet therapy such lenalidomide and dexamethasone.^197^

### 8.5 Treatment of relapsed or refractory myeloma

The majority of MM patients is relapse. This is heralded by a rise in serum M‐protein and/or light chains. Treatment should be instigated with the rapid increase in myeloma biochemical parameters or at the onset of end‐organ damage. Patients should therefore be monitored with regular assessment including measurement of myeloma biochemical parameters, and in in the absence of a biomarker, regular imaging. At relapse, the MM tumor contains significantly more mutations than the primary tumor sample.^105^ Furthermore, clonal selection of mutations occur at relapse and are accompanied by subclonal heterogeneity.^105^ This represents a significant therapeutic challenge.

At the time of relapse, the treatment choice is affected by patient‐related and disease‐related factors. These factors include patient preference, age, cytogenetic profile, pre‐existing toxicities, comorbidities, relapse characteristics, and by the type of, and the response to, previous therapies.^198^, ^199^ A change to, or the addition of, a class of drug the patient has not previously been exposed to is generally warranted at each relapse.^198^, ^199^ An approach to the management of MM at first relapse, along with examples of treatment regimens used is provided in Figure S4. As the number of lines of therapy increases, the time to progression and depth of response decreases.^199^ The outlook is poor for patients refractory to proteasome inhibitors, immunomodulatory agents and anti‐CD38 antibodies with a median overall survival of 5.6 months.^200^ For such individuals, enrolment on a clinical trial is recommended. In the absence of a clinical trial, dependent on approval and access, novel approaches such as nuclear export inhibition,^201^ pan‐deacetylase inhibition,^202^ an anti‐SLAMF7 antibody,^203^ bispecific T‐cell engaging antibodies,^204^ antibody‐drug conjugates^205^ and chimeric antigen receptor T‐cell therapy are currently being used.^206^ Loss of targeted antigens (such as BCMA) and soluble circulating antigens represent a challenge to targeted immunotherapies which may be overcome by γ‐secretase inhibitors and targeting multiple antigens,^207^, ^208^, ^209^ whereas venetoclax (a BCL2 inhibitor) in combination with bortezomib in relapsed/refractory MM results in improved progression‐free survival, the development of treatment‐emergent fatal infections led to the trial closing early.^210^ A subgroup analysis suggests t(11;14) MM have an improved progression‐free survival without reduced survival and response may be predicted by a high BCL2/BCL2L1 expression ratio.^211^ Such findings require larger prospective trials.

### 8.6 Supportive care of myeloma

With an incidence of 20% to 40%, renal dysfunction is a common complication in MM and is associated with significant morbidity and mortality.^11^, ^199^ Renal dysfunction is multifactorial with common causes including light‐chain cast nephropathy, dehydration, hypercalcemia and less common causes such as amyloidosis and a plasma cell infiltrate.^212^, ^213^ Also, the optimization of renal function through treatment of infection, avoidance of dehydration and nephrotoxic drugs, antimyeloma therapy in the form of proteasome inhibitors and immunomodulatory agents improve renal function and overall survival.^214^ Patients presenting acute renal failure requiring dialysis have a higher probability of renal function recovery and independence from dialysis if a rapid disease response is achieved.^215^ Bortezomib‐based triplet therapy offers high rates of myeloma response and subsequent renal response.^216^

Damage to the structure of bone itself is a major cause of morbidity in MM. Malignant plasma cells secrete osteoclast‐activating and osteoblast‐inhibitory factors leading to bone resorption.^217^, ^218^ Intravenous bisphosphonates or denosumab is initiated in patients with MM requiring therapy due to the efficacy in preventing skeletal‐related events.^219^, ^220^ Osteonecrosis of the jaw and atypical femoral fractures are recognized significant complications. Radiotherapy can be used to mitigate pain secondary to bone lesions and prevent fractures.^20^ Surgery may be required to prevent or treat fractures as well as improve pain with significant vertebral bone disease.^221^

Patients with plasma cell dyscrasia are at increased risk of thrombotic complications due to patient related factors, underlying disease and treatment.^222^, ^223^ Patients receiving immunomodulatory agents are particularly susceptible to thrombosis and require aspirin, low‐molecular heparin or a direct oral anticoagulant, dependent risk stratification.^224^, ^225^

Infection is a significant cause of morbidity and mortality in patients with MM. The highest risk in the first 3 months of induction therapy.^226^, ^227^ Three months of levofloxacin at induction is recommended with induction therapy.^228^ Additional antimicrobial prophylaxis in the form of acyclovir, fluconazole and trimethoprim‐sulfamethoxazole is used but is treatment and center dependent.^229^ Patients with MM are at significant risk of morbidity and mortality from SARS‐CoV‐2 viral infection.^230^, ^231^ Monitoring and treating patients with MM during the pandemic has therefore required adaptation.^232^ Whereas the development and approval of vaccines will reduce transmission and risk of severe COVID‐19, there are concerns that patients with MM, particularly those with active disease, receiving treatment or with immunoparesis will not generate an appropriate antibody response.^233^, ^234^, ^235^

## 9 CONCLUSION

Improvements in diagnostics, risk stratification, treatment and supportive care have led to an increase in overall survival in MM over recent decades. The increase in the number of people living with MM requires further work on the identification and management of cumulative disease and treatment‐related healthcare burdens.^236^ So far, only a minority of clinical trials in MM over the past 15 years use overall survival or health‐related quality of life as a primary endpoint.^237^

Despite improvements in care, MM remains incurable and the majority of patients succumb to their disease. As well as optimizing the sequencing of existing therapies, novel therapies are required particularly for patients who are refractory to approved drugs, for those with poor performance status and those with high‐risk MM (with a median OS of <2 years) from diagnosis). For example, new approaches which utilize the immune system to exert anti‐myeloma effects are becoming central to the management of MM.

Clonal heterogeneity and clonal evolution limit the prospect of genetically informed targeted therapies, in isolation, offering significant clinical benefit for a large number of patients with MM. Improving our understanding of the biological consequences of genetic susceptibility, somatic mutations and the mechanisms underlying clonal evolution represents one approach to realizing the potential of genetic studies of MM. Clonal evolution of MGUS and its progression to MM takes decades, offering a window for early intervention before the life‐threatening disease arises. MGUS is a common condition but has remained in the periphery of hematological research.

Access to drugs is currently costly which limits access in low‐income and middle‐income countries many of which have limited access to existing effective antimyeloma medications and have an increased mortality rate from MM. In high‐income countries, high‐cost drugs raise questions regarding the value of healthcare.

## CONFLICT OF INTEREST

The authors declare no conflicts of interest.

## Supporting information


**Appendix S1** Supporting Information
