# Supplementary material for: Epidemiology, genetics and treatment of multiple myeloma and precursor diseases
Source: Int J Cancer. 2021 Aug 30;149(12):1980–96. doi: 10.1002/ijc.33762 (PMC11497332; doi:10.1002/ijc.33762)
Supplement: Supplementary file 1 — Appendix S1 Supporting Information [file IJC-149-1980-s001.pdf]

Epidemiology, genetics and treatment of multiple myeloma and precursor diseases

Kari Hemminki, Asta Försti, Richard Houlston and Amit Sud

SUPPLEMENTARY MATERIAL

Table of contents:

Supplementary Table 1: Myeloma risks for offspring and siblings with affected family members (from ref. 49)

Supplementary Figure 1: Incidence (from 1960, solid lines) and mortality (from 1953, broken lines) in multiple myeloma in Sweden through 2016

Supplementary Figure 2: Meta-analysis of genome-wide association studies from international multiple myeloma cohorts (7319 MM patients) as described and annotated by Went et al. (ref. 59)

Supplementary Figure 3: Genes annotated by germline susceptibility and recurrent somatic mutations in MM

Supplementary Figure 4: An approach to the management of patients with MM at first relapse

Supplementary Table 1: Myeloma risks for offspring and siblings with affected family members (from ref. 49).

Cancer in family member[1)]	Affected Parent			Affected Sibling		
	**Cases**	**RR**	**95% CI**	**Cases**	**RR**	**95% CI**
Esophagus	13	0.76	(0.44-1.31)	12	**1.84**[3)]	(1.02-3.33)
**Colorectum**[2)]	284	**1.18**	(1.04-1.34)	106	**1.47**	(1.20-1.79)
**Breast**	217	1.05	(0.85-1.30)	171	**1.27**	(1.02-1.58)
**Prostate**	327	**1.15**	(1.03-1.29)	140	1.17	(0.99-1.39)
Nervous system	55	1.11	(0.87-1.42)	47	**1.40**	(1.07-1.82)
Endocrine glands	42	**1.40**	(1.02-1.94)	21	1.13	(0.66-1.96)
Bone	4	1.50	(0.43-5.21)	4	2.01	(0.47-8.62)
Lower extremity	2	3.19	(0.70-14.55)	3	**4.18**	(1.18-14.79)
Connective tissue	23	**1.83**	(1.20-2.79)	8	1.32	(0.63-2.76)
Upper extremities	5	**2.85**	(1.19-6.82)	0		
Retroperitoneum	6	**3.32**	(1.43-7.70)	0		
**Myeloma**	67	**2.03**	(1.60-2.58)	30	**2.90**	(2.04-4.14)
**Leukemia**	76	**1.34**	(1.06-1.70)	24	1.11	(0.73-1.69)
**CLL**	36	**1.67**	(1.20-2,32)	8	1.02	(0.51-2.03)
Myelofibrosis	3	1.08	(0.38-3.06)	5	**2.45**	(1.02-5.92)
Cancer of unknown primary	91	**1.23**	(1.01-1.50)	24	1.12	(0.76-1.65)

RR, relative risk; CI, confidence interval

[1)] Bolding of the name of the cancer indicates that the result was also significant for the particular cancer when a parent was diagnosed with myeloma (i.e., independent analysis, see ref. 49).
[2)] The RR was 1.91 (N=15, 95%CI 1.14-3.19) when a parent and a sibling were diagnosed with colorectal cancer.
[3)] Bold of RR indicates that the 95%CIs do not include 1.00.

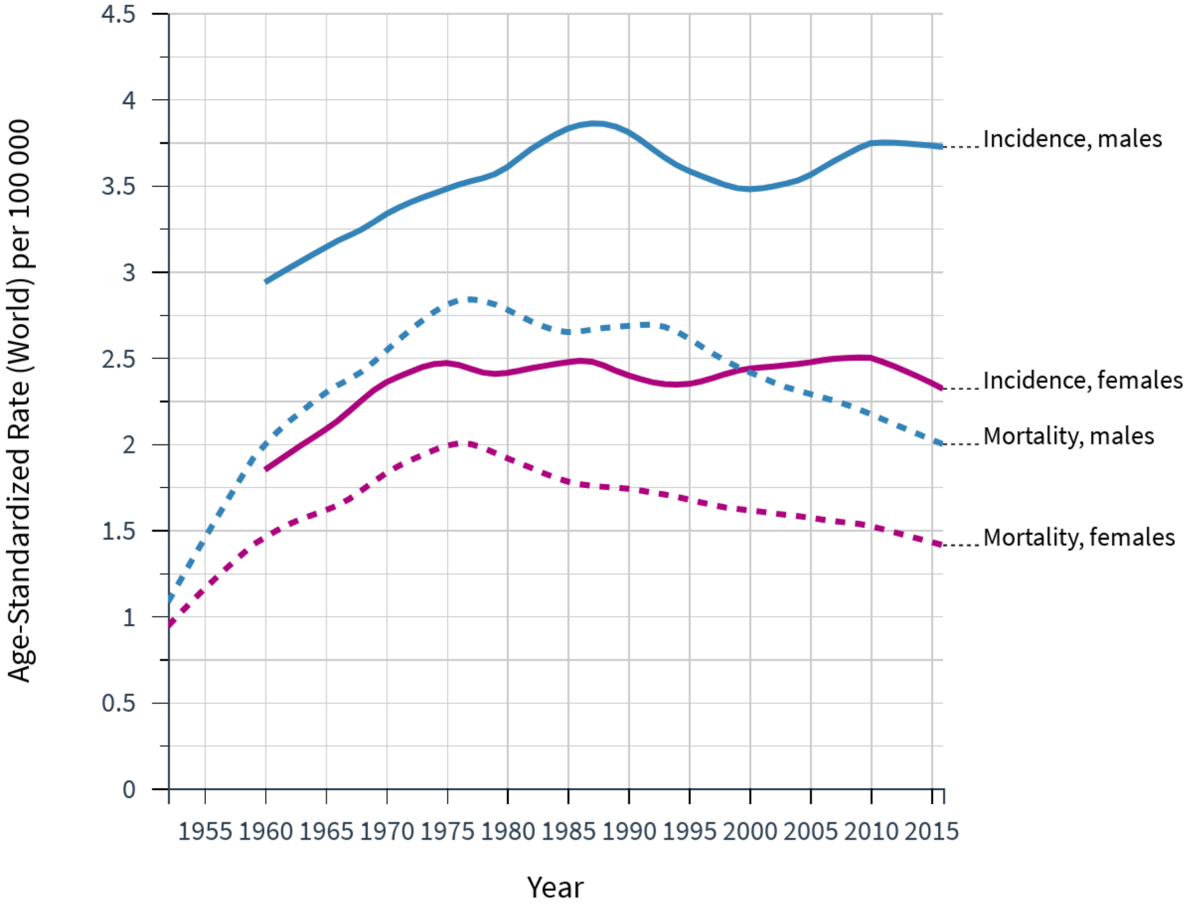

Supplementary Figure 1: Incidence (from 1960, solid lines) and mortality (from 1953, broken lines) in multiple myeloma in Sweden through 2016. Lines corresponding to men are blue whereas lines corresponding to women are magenta. The rates are adjusted to the world standard population. The data are from the Nordcan database from the International Agency for Research on Cancer.

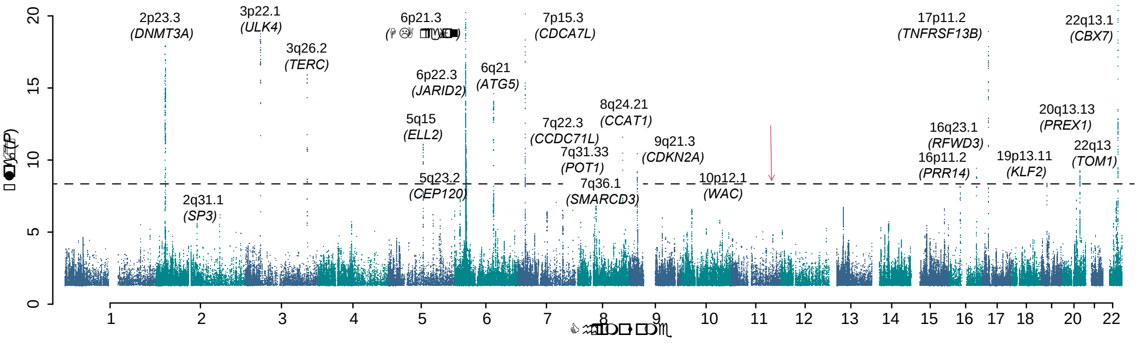

Supplementary Figure 2: Meta-analysis of genome-wide association studies from international multiple myeloma cohorts (7319 MM patients) as described and annotated by Went et al. (ref. 59) The Manhattan plot shows the genomic location of each SNP on the x-axis and their association P-values on the y-axes. The broken line depicts the genome-wide P-value of $5x10^{-8}$. The *CCND1* locus which is associated with the translocation (11;14) is marked by an arrow.

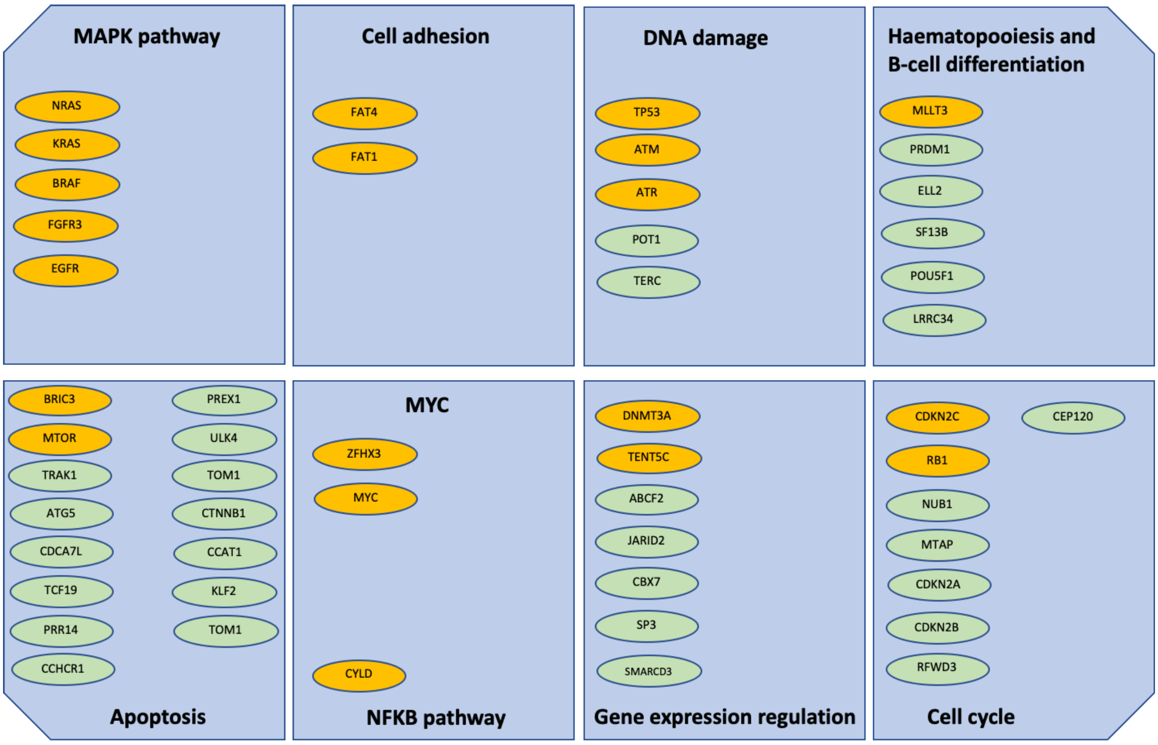

Supplementary Figure 3: Genes annotated by germline susceptibility and recurrent somatic mutations in MM. Genes in green ellipsoids represent genes implicated in genetic susceptibility whereas genes in yellow ellipsoids represent the most common recurrent coding somatic mutations.

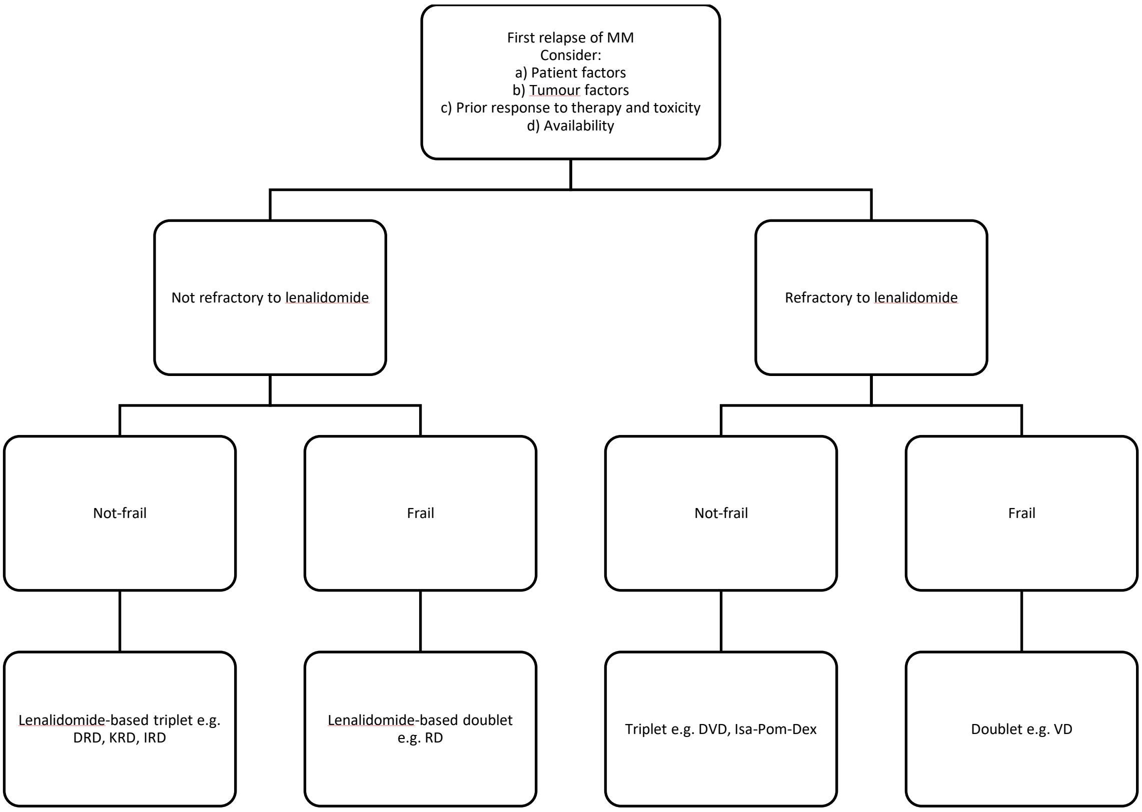

Supplementary Figure 4: An approach to the management of patients with MM at first relapse. MM, multiple myeloma; DRD, daratumumab, lenalidomide, dexamethasone; KRD, carfilzomib, lenalidomide, dexamethasone; IRD, ixazomib, lenalidomide, dexamethasone ; RD, lenalidomide, dexamethasone; DVD, daratumumab, bortezomib, dexamethasone; Isa-Pom-Dex, isatuximab, pomalidomide, dexamethasone; VD, bortezomib, dexamethasone. A guide to the current approach and possible regimens used to treat multiple myeloma at first relapse.
